# Antidepressants available in Japan for older people with major depressive disorder: A systematic review and meta‐analysis

**DOI:** 10.1002/npr2.12422

**Published:** 2024-02-06

**Authors:** Taro Kishi, Kenji Sakuma, Masakazu Hatano, Takenori Okumura, Masaki Kato, Hajime Baba, Nakao Iwata

**Affiliations:** ^1^ Department of Psychiatry Fujita Health University School of Medicine Toyoake Aichi Japan; ^2^ Department of Pharmacotherapeutics and informatics Fujita Health University School of Medicine Toyoake Aichi Japan; ^3^ Department of Neuropsychiatry Kansai Medical University Osaka Japan; ^4^ Department of Psychiatry Juntendo University Koshigaya Hospital Saitama Japan; ^5^ Department of Psychiatry and Behavioral Science Juntendo University Graduate School of Medicine Tokyo Japan

**Keywords:** antidepressants available in Japan, efficacy, older people with major depressive disorder, safety, systematic review and meta‐analysis

## Abstract

**Aim:**

To update the major depressive disorder (MDD) treatment guidelines of the Japanese Society of Mood Disorders, we conducted a systematic review and pairwise meta‐analysis of double‐blind, randomized, placebo‐controlled trials of available antidepressants in Japan for older adults with MDD.

**Methods:**

Outcome measures included response rate (primary), improvement in depressive symptom scale score, remission rate, all‐cause discontinuation, discontinuation due to adverse events, and at least one adverse event. A random‐effects model was used to calculate the risk ratio (RR) and standardized mean difference (SMD) with a 95% confidence interval (95% CI).

**Results:**

Nine double‐blind, randomized, placebo‐controlled trials (*n* = 2145) were identified. No study has been conducted in Japan. Our meta‐analysis included the following antidepressants: duloxetine, escitalopram, imipramine, sertraline, venlafaxine, and vortioxetine. Antidepressants have significantly higher response rates than placebo (RR [95% CI] = 1.38 [1.04, 1.83], *p* = 0.02). Antidepressants outperformed placebo in terms of improving depressive symptom scale score (SMD [95% CI] = −0.62 [−0.92, −0.33], *p* < 0.0001). However, antidepressants were associated with a higher discontinuation rate due to adverse events (RR [95% CI] = 1.94 [1.30, 2.88], *p* = 0.001) and a higher incidence of at least one adverse event (RR [95% CI] = 1.11 [1.02, 1.21], *p* = 0.02) compared to placebo. The groups did not differ significantly in terms of remission rate or all‐cause discontinuation.

**Conclusions:**

Our meta‐analysis concluded that treatment with antidepressants available in Japan is only weakly recommended for moderate to severe MDD in older adults.

## Abstract 2

Our meta‐analysis revealed that antidepressants available in Japan were more effective in treating moderate to severe older people with major depressive disorder. However, antidepressants were poorly tolerated in those people. Thus, treatment with antidepressants available in Japan is only weakly recommended for moderate to severe older people with major depressive disorder.

## Graphical abstract

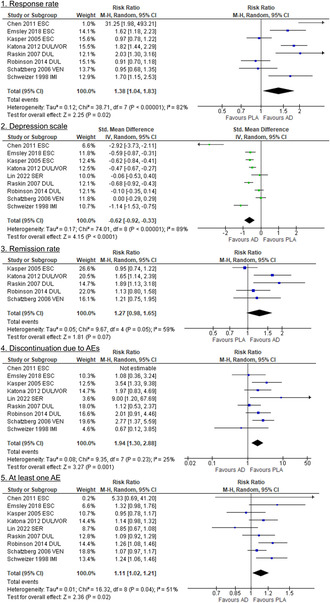

## 1 INTRODUCTION

Recent treatment guidelines have shed light on the etiology, clinical course, and prognosis of adults with early‐ and late‐onset depression.^1^ A network meta‐analysis for general adults (approximately <65 years) with major depressive disorder (MDD) found that all antidepressants were associated with higher response rates compared to placebo.^2^ However, a network meta‐analysis for older adults (approximately ≥65 years) with MDD (O‐MDD) revealed that the majority of antidepressants had similar response rates to placebo (only duloxetine was effective).^3^ A recent pairwise meta‐analysis also found that pooled newer antidepressants were not more effective for O‐MDD than placebo.^4^ However, this meta‐analysis included antidepressants unavailable in Japan, such as buspirone, citalopram, and fluoxetine. Furthermore, some double‐blind, randomized, placebo‐controlled trials (DBRPCTs) were published after previous meta‐analyses^3^, ^4^, ^5^ were completed. To update the MDD treatment guidelines of the Japanese Society of Mood Disorders,^6^ we conducted a systematic review and pairwise meta‐analysis using DBRPCTs of available antidepressants in Japan for O‐MDD.

## 2 METHODS

This study followed the Preferred Reporting Items for Systematic Reviews and Meta‐Analyses guidelines (Table S1).^7^ The detailed methods are provided in the Open Science Framework (https://osf.io/vnx2u). Detailed information about our literature search strategy is shown in Figure S1. In summary, a literature search of previous systematic reviews was conducted until 2016–2017 using the same inclusion criteria as our systematic review.^3^, ^4^, ^5^ To update the literature search of previous systematic reviews,^3^, ^4^, ^5^ we searched the PubMed, Cochrane Library, and Embase databases for relevant studies published between January 1, 2016, and November 23, 2023. The PICO strategy for our study is as follows:

P: O‐MDD
I: Pooled available antidepressants in Japan
C: Placebo
O: Response rate (primary), improvement in depressive symptom scale score, remission rate, all‐cause discontinuation, discontinuation due to adverse events, and at least one adverse event (data synthesis for efficacy outcomes: Table S2). If the required data was missing from the studies, we looked for it in published systematic review articles.

A random‐effects model was used to calculate the risk ratio (RR) and standardized mean difference (SMD) with a 95% confidence interval (95% CI). Heterogeneity was assessed using *I* ^2^ statistics (*I* ^2^ ≥ 50% indicating heterogeneity).^8^ In the event of significant differences in the dichotomous variable between treatment groups, the number needed to treat to benefit (NNTB) or harm (NNTH) was estimated using the exact rates and 95% CIs in each outcome in both the antidepressant and placebo groups, as determined by a single‐group summary meta‐analysis. Because the primary outcome results showed significant heterogeneity (*I* ^2^ = 82%), we conducted sensitivity and meta‐regression analyses to look for any confounding factors. Furthermore, because imipramine has been shown to have a higher risk of various adverse events when compared to newer antidepressants,^9^, ^10^ another sensitivity analysis was performed for all‐cause discontinuation, discontinuation due to adverse events, and at least one adverse event, excluding imipramine's DBRPCT.^10^ Egger's regression was used to identify publication bias in the meta‐analysis. These analyses were conducted using Review Manager software (version 5.4)^8^ and Comprehensive Meta‐Analysis Software Version 3 (Biostat Inc., Englewood, NJ, USA).

## 3 RESULTS

**FIGURE 1:**
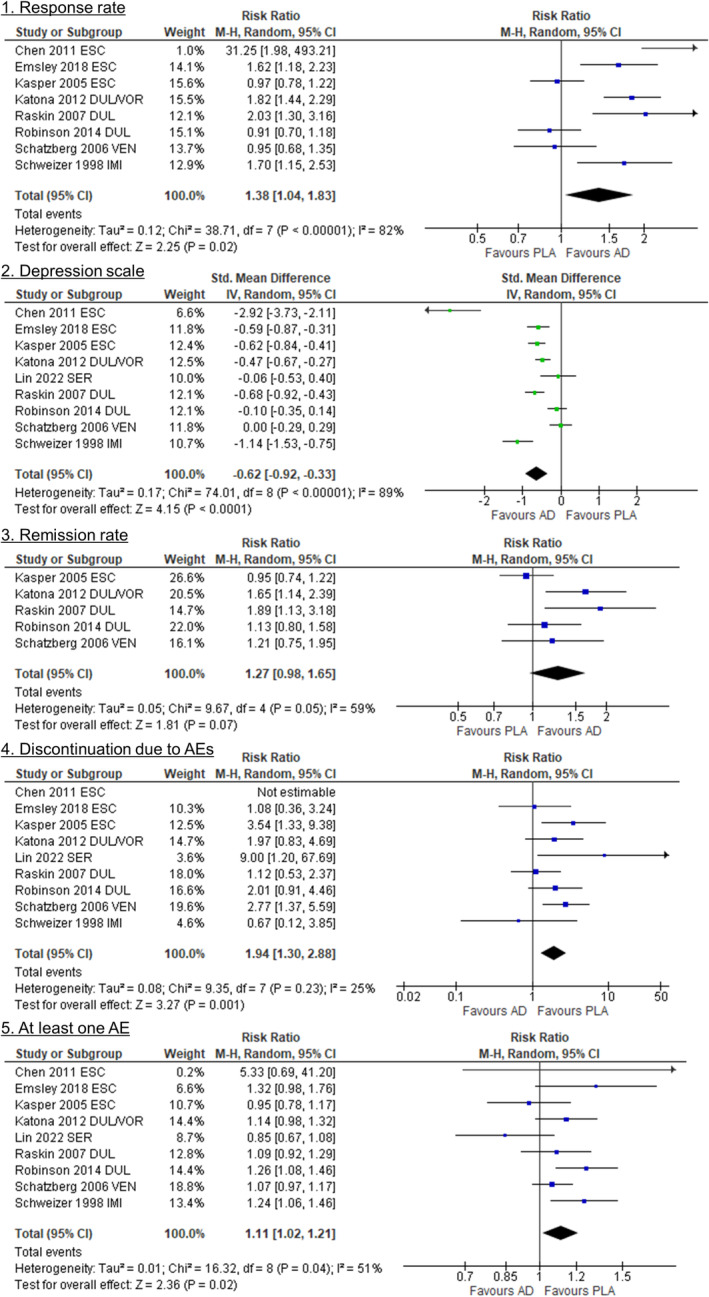
Forest plots. 1. Response rate; 2. Depression scale; 3. Remission rate; 4. Discontinuation due to AEs; At least one AE. 95% CI, 95% confidence interval; AD, antidepressant; AE, adverse event; DUL, duloxetine; ESC, escitalopram; IMI, imipramine; PLA, placebo; SER, sertraline; Std. Mean Differences, standardized mean difference; VEN, venlafaxine; VOR, vortioxetine.

The literature search results are displayed in Figure S1. Nine DBRPCTs (*n* = 2145) were identified.^10^, ^11^, ^12^, ^13^, ^14^, ^15^, ^16^, ^17^, ^18^ The study characteristics included in our meta‐analysis are summarized in Table S3. Regarding the inclusion criteria on age, the Lin 2022 study was 55 years or older (mean age of this study: 68.7 ± 7.4 years),^15^ while the other studies were 65 years or older. According to the depression scale scores at baseline in each trial, the participants' illness severity was considered moderate to severe. Our meta‐analysis included the following antidepressants: duloxetine, escitalopram, imipramine, sertraline, venlafaxine, and vortioxetine. Individual studies produced inconsistent efficacy outcomes (Table S2). For the overall risk of bias,^8^ the Chen 2011 study was rated as high risk,^11^ the other four studies as having some concerns, and the rest as low risk (Figure S2). Treatment with antidepressants resulted in significantly more responders than placebo (RR [95% CI] = 1.38 [1.04, 1.83], *p* = 0.02, *I* ^2^ = 82%, NNTB [95% CI] = 7 [4, 16], Figure 1). The accurate response rates (95% CI) in the antidepressant and placebo groups were 50.9% (42.5%, 59.3%) and 36.1% (28.6%, 44.4%), respectively. We confirmed that there was no publication bias for the response rate (Figure S3). Antidepressants outperformed placebo in improving the depressive symptom scale score (SMD [95% CI] = −0.62 [−0.92, −0.33], *p* < 0.0001, *I* ^2^ = 89%, Figure 1). However, antidepressants were associated with higher discontinuation due to adverse events compared to placebo (RR [95% CI] = 1.94 [1.30, 2.88], *p* = 0.001, *I* ^2^ = 25%, NNTH [95% CI] = 20 [10, 63], Figure 1). The accurate discontinuation rates due to adverse events (95% CI) in the antidepressant and placebo groups were 10.8% (7.3%, 15.8%) and 5.7% (4.2%, 7.7%), respectively. Furthermore, antidepressants were linked to a higher risk of at least one adverse event compared to placebo (RR [95% CI] = 1.11 [1.02, 1.21], *p* = 0.02, *I* ^2^ = 51%, NNTH = not significant, Figure 1). The remission rate and all‐cause discontinuation were not significantly different between the groups (Figure 1 and Figure S4).

The sensitivity analyses (antidepressant class and overall risk of bias) revealed no subgroups with a substantially reduced heterogeneity (Table S4). Our meta‐regression analyses also revealed no associations between any of the factors (mean age, percentage of males, antidepressant dose, dosing schedule, study duration, and total participants) and the effect size for response rate (Table S5). Another sensitivity analysis, which excluded the imipramine study revealed that, while newer antidepressants were still associated with a higher discontinuation rate due to adverse events, there was no significant difference in the incidence of at least one adverse event between newer antidepressants and placebo (Table S6).

## 4 DISCUSSION

Our meta‐analysis revealed that antidepressants available in Japan were more effective in treating moderate to severe O‐MDD. However, the efficacy outcome results showed considerable heterogeneity. We were also unable to identify any causes of the heterogeneities. Furthermore, antidepressants were poorly tolerated in patients with O‐MDD. Because our recent network meta‐analysis revealed that the safety profile of each antidepressant varies greatly,^9^ clinicians should exercise extreme caution when treating physical and mental aspects of O‐MDD, including prescribing antidepressants. Thus, treatment with antidepressants available in Japan is only weakly recommended for moderate to severe O‐MDD.

Our study had several limitations. First, because there were few studies and participants. Second, our study did not cover all antidepressants available in Japan, including fluvoxamine, milnacipran, mirtazapine, and paroxetine. Third, we did not assess the efficacy, acceptability, tolerability, or safety of individual antidepressants in treating O‐MDD. Fourth, while our meta‐analysis focused on antidepressants available in Japan, it did not include any studies conducted in Japan. As a result, our meta‐analysis findings may not be directly applicable to the Japanese population.

## AUTHOR CONTRIBUTIONS

TK developed the study concept and design, and performed the statistical analyses. TK and SK took full responsibility for the data integrity and the accuracy of the data analysis. All authors interpreted the data and wrote the article. NI supervised the article.

## FUNDING INFORMATION

This work was supported by JSPS KAKENHI Grant Number 23K06998.

## CONFLICT OF INTEREST STATEMENT

The authors have no specific conflicts of interest to declare concerning this study. They disclose the following interests that have arisen in the last 3 years: TK has received speaker's honoraria from Eisai, Janssen, Meiji, MSD, Otsuka, Sumitomo, Takeda, Mitsubishi‐Tanabe, Yoshitomi, Kyowa, and Viatris and research grants from Eisai, Grant‐in‐Aid for Scientific Research (19K08082 and 23K06998), Japan Agency for Medical Research and Development (JP22dk0307107, JP22wm0525024, JP23dk0307117, JP23wm0525024, and JP23dk0307122), and the Japanese Ministry of Health, Labour and Welfare (21GC1018). KS has received speaker's honoraria from Eisai, Janssen, Kyowa, Meiji, Otsuka, Sumitomo, and Takeda and research grants from Grant‐in‐Aid for Young Scientists (19K17099), Grant‐in‐Aid for Scientific Research © (23K06998), Fujita Health University School of Medicine Research Grant for Early‐Career Scientists, and Japan Agency for Medical Research and Development (JP22dk0307107). TO has received speaker's honoraria from Meiji, Kowa, Otsuka, Sumitomo, and Eisai. MH has received speaker's honoraria from Meiji, Sumitomo, and WELCIA, and Grant‐in‐Aid for Early‐Career Scientists (23K14827). MK received consulting fees from Sumitomo, Otsuka, Lundbeck, Takeda, and Shionogi; payment/honoraria from Sumitomo, Otsuka, Meiji, Eli Lilly, MSD, Pfizer, Janssen, Shionogi, Mitsubishi‐Tanabe, Takeda, Lundbeck, Viatris, Eisai, Kyowa, and Ono; and has received grant funding from the Japan Society for the Promotion of Science (22K07607), Japan Agency for Medical Research and Development (JP20dk0307081), SENSHIN Medical Research Foundation, the Japan Research Foundation for Clinical Pharmacology, and the Japanese Society of Clinical Neuropsychopharmacology. HB received grant funding from the Japan Society for the Promotion of Science and Esai and speaker's honoraria from Otsuka, Sumitomo, MSD, Meiji, Pfizer, Yoshitomi, Janssen, Kyowa, Mitsubishi‐Tanabe, Viatris, Takeda, Esai, Lundbeck, Mylan EPD, Mochida, Sawai, Kowa, Towa, and EA Pharma. NI has received speaker's honoraria from Eisai, Janssen, Meiji, Otsuka, Sumitomo, Takeda, Tanabe‐Mitsubishi, and Viatris and research grants from Daiichi Sankyo, Eisai, Meiji, Otsuka, Sumitomo, Takeda, and Mitsubishi‐Tanabe.

## ETHICS STATEMENT

Approval of the research protocol by an Institutional Reviewer Board: N/A.

Informed Consent: N/A.

Registry and the Registration No. of the study/trial: N/A.

Animal Studies: N/A.

## Supporting information


Appendix S1

## Data Availability

All descriptive variables are openly available in the articles of the studies that are cited in this article and ClinicalTrials.gov (https://clinicaltrials.gov/).
