# Supplementary material for: Antidepressants available in Japan for older people with major depressive disorder: A systematic review and meta‐analysis
Source: Neuropsychopharmacol Rep. 2024 Feb 6;44(1):267–71. doi: 10.1002/npr2.12422 (PMC10932767; doi:10.1002/npr2.12422)
Supplement: Supplementary file 1 — Appendix S1 [file NPR2-44-267-s001.pdf]

Figure S1. Preferred Reporting Items for Systematic reviews and Meta-Analyses flow diagram.

a literature search of previous systematic reviews (Gutsmiedl 2020, Krause 2019, Mallery 2019) was conducted until 2016–2017 using the same inclusion criteria as our systematic review. To update the literature search of previous systematic reviews (Gutsmiedl 2020, Krause 2019, Mallery 2019), we searched the PubMed, Cochrane Library, and Embase databases for relevant studies published between January 1, 2016, and November 23, 2023. The search terms for PubMed included (late-life [tiab] OR late life [tiab] OR older [tiab] OR elder [tiab] OR elderly [tiab]) AND (placebo [tiab]) AND (randomized OR random OR randomly) AND (major depression OR major depressive disorder). The search terms for the Cochrane Library included (late-life OR late life OR older OR elder OR elderly) AND (placebo) AND (randomized OR random OR randomly) AND (major depression OR major depressive disorder). This search used "Title Abstract Keyword mode." The search terms for Embase included ('placebo'/exp OR placebo) AND ('randomized controlled trial'/exp OR 'randomized controlled trial') AND ('older adults'/exp OR 'older adults' OR elderly) AND ('major depression'). No language restriction was employed to the literature search. The search terms for ICHUSHI (Japanese) included (elderly) AND (antidepressant) AND (randomized OR double-blind). The authors assessed the retrieved studies and selected those that were eligible. Moreover, we manually searched the reference lists of the included studies and reviews for additional relevant published and unpublished research, including conference abstracts. We also searched clinical trial registries, such as ClinicalTrials.gov (http://clinicaltrials.gov/) and the World Health Organization International Clinical Trials Registry Platform (http://www.who.int/ictrp/search/en/) to ensure that the included set of DBRPCTs was comprehensive and to minimize the influence of publication bias. Any discrepancies in the article selection were addressed via a consensus among the authors.

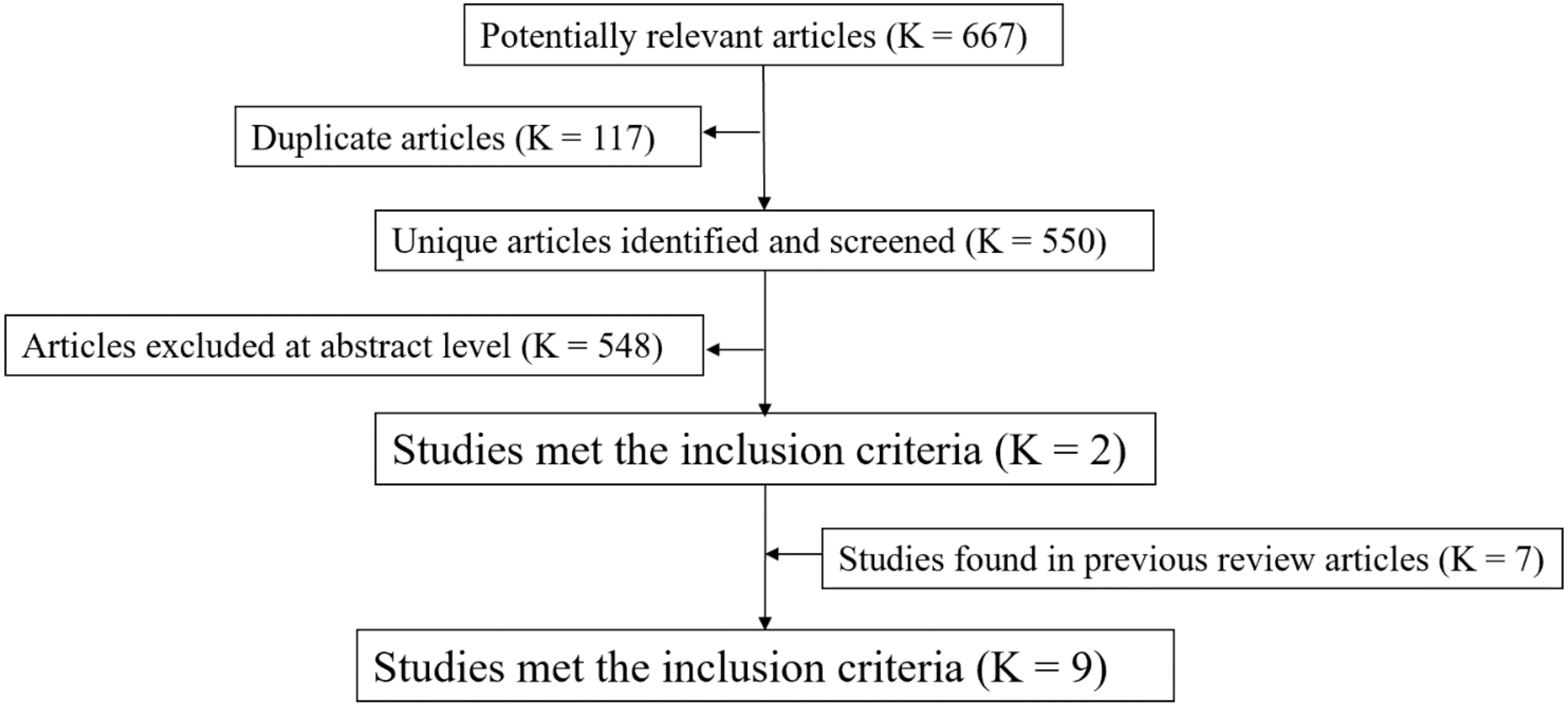

Review articles that we read for the literature search.

1. Gutsmiedl K, Krause M, Bighelli I, Schneider-Thoma J, Leucht S. How well do elderly patients with major depressive disorder respond to antidepressants: a systematic review and single-group meta-analysis. BMC Psychiatry 2020; 20: 102.
2. Krause M, Gutsmiedl K, Bighelli I, Schneider-Thoma J, Chaimani A, Leucht S. Efficacy and tolerability of pharmacological and non-pharmacological interventions in older patients with major depressive disorder: A systematic review, pairwise and network meta-analysis. Eur Neuropsychopharmacol 2019; 29: 1003-1022.
3. Mallery L, MacLeod T, Allen M et al. Systematic review and meta-analysis of second-generation antidepressants for the treatment of older adults with depression: questionable benefit and considerations for frailty. BMC Geriatr 2019; 19: 306.

Articles included in our systematic review (underlined studies were detected by our literature search).

1. Chen YM, Huang XM, Thompson R, Zhao YB. Clinical features and efficacy of escitalopram treatment for geriatric depression. J Int Med Res 2011; 39: 1946-53.
2. <u>Emsley R, Ahokas A, Suarez A et al. Efficacy of Tianeptine 25-50 mg in Elderly Patients With Recurrent Major Depressive Disorder: An 8-Week Placebo- and Escitalopram-Controlled Study. J Clin Psychiatry 2018; 79.</u>
3. Kasper S, de Swart H, Friis Andersen H. Escitalopram in the treatment of depressed elderly patients. Am J Geriatr Psychiatry 2005; 13: 884-91.
4. Katona C, Hansen T, Olsen CK. A randomized, double-blind, placebo-controlled, duloxetine-referenced, fixed-dose study comparing the efficacy and safety of Lu AA21004 in elderly patients with major depressive disorder. Int Clin Psychopharmacol 2012; 27: 215-23.
5. <u>Lin CH, Wang SH, Lane HY. Effects of Sodium Benzoate, a D-Amino Acid Oxidase Inhibitor, on Perceived Stress and Cognitive Function Among Patients With Late-Life Depression: A Randomized, Double-Blind, Sertraline- and Placebo-Controlled Trial. Int J Neuropsychopharmacol 2022; 25: 545-555.</u>
6. Raskin J, Wiltse CG, Siegal A et al. Efficacy of duloxetine on cognition, depression, and pain in elderly patients with major depressive disorder: an 8-week, double-blind, placebo-controlled trial. Am J Psychiatry 2007; 164: 900-9.
7. Robinson M, Oakes TM, Raskin J, Liu P, Shoemaker S, Nelson JC. Acute and long-term treatment of late-life major depressive disorder: duloxetine versus placebo. Am J Geriatr Psychiatry 2014; 22: 34-45.
8. Schatzberg A, Roose S. A double-blind, placebo-controlled study of venlafaxine and fluoxetine in geriatric outpatients with major depression. Am J Geriatr Psychiatry 2006; 14: 361-70.
9. Schweizer E, Rickels K, Hassman H, Garcia-Espana F. Buspirone and imipramine for the treatment of major depression in the elderly. J Clin Psychiatry 1998; 59: 175-83.

Study that the previous systematic review included, but this was not included in the current systematic review.

1. EUCTR003821-25- DK 2005 (because we did not obtain the sufficient data for performing the systematic review)

NCT00130455 (because we did not obtain the sufficient data for performing the systematic review)

Figure S2. Risk of bias summary.

	Randomization process	Deviation from intended intervention	Missing outcome data	Measurement of the outcome	Selection of the reported result	Overall risk of bias
Chen 2011	Some concerns	Some concerns	High risk	Low risk	Low risk	High risk
Emsley 2018	Low risk	Low risk	Low risk	Low risk	Low risk	Low risk
Kasper 2005	Some concerns	Some concerns	Low risk	Some concerns	Low risk	Some concerns
Katona 2012	Low risk	Low risk	Low risk	Low risk	Low risk	Low risk
Lin 2022	Some concerns	Some concerns	Low risk	Low risk	Low risk	Some concerns †
Raskin 2007	Some concerns	Low risk	Low risk	Low risk	Low risk	Some concerns
Robinson 2014	Low risk	Low risk	Low risk	Low risk	Low risk	Low risk
Schatzberg 2006	Low risk	Low risk	Low risk	Low risk	Low risk	Low risk
Schweizer 1998	Some concerns	Some concerns	Low risk	Low risk	Low risk	Some concerns

Version 2 of the Cochrane risk-of-bias tool for randomized trials (https://www.riskofbias.info/)

†Dose range was 25–150 mg/day. However, final mean dose: 66.7±30.5 mg/d

Figure S3. Funnel plot of the response rate.

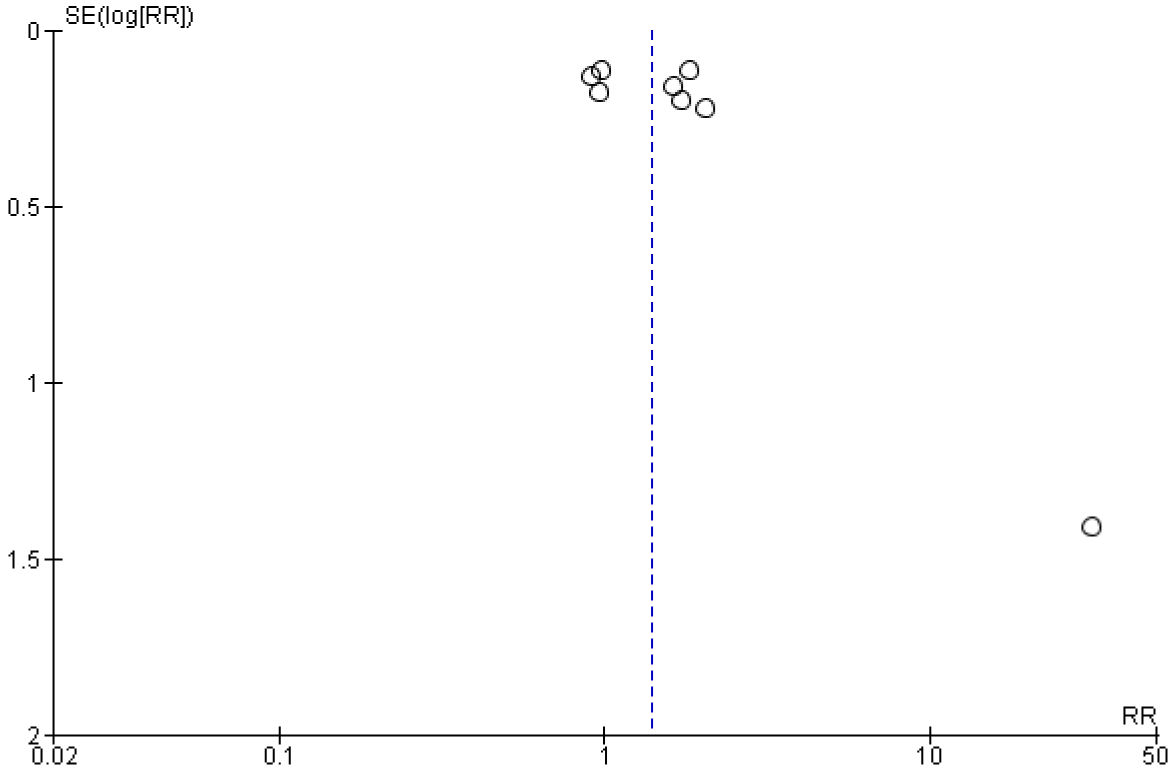

Egger regression test.

p = 0.153

Figure S4. Forest plot for all-cause discontinuation.

Study or Subgroup	Weight	Risk Ratio M-H, Random, 95% CI
Chen 2011 ESC	0.9%	0.90 [0.14, 5.92]
Emsley 2018 ESC	6.7%	0.76 [0.38, 1.52]
Kasper 2005 ESC	10.8%	1.51 [0.89, 2.56]
Katona 2012 DUL/VOR	10.9%	1.19 [0.71, 2.02]
Lin 2022 SER	7.1%	1.50 [0.77, 2.92]
Raskin 2007 DUL	15.2%	0.94 [0.61, 1.46]
Robinson 2014 DUL	26.0%	0.79 [0.58, 1.08]
Schatzberg 2006 VEN	15.2%	1.38 [0.90, 2.14]
Schweizer 1998 IMI	7.1%	1.08 [0.55, 2.09]
**Total (95% CI)**	**100.0%**	**1.06 [0.88, 1.27]**
Total events		

Heterogeneity: $Tau^2 = 0.01$; $Chi^2 = 8.98$, df = 8 (P = 0.34); $I^2 = 11\%$

Test for overall effect: Z = 0.62 (P = 0.54)

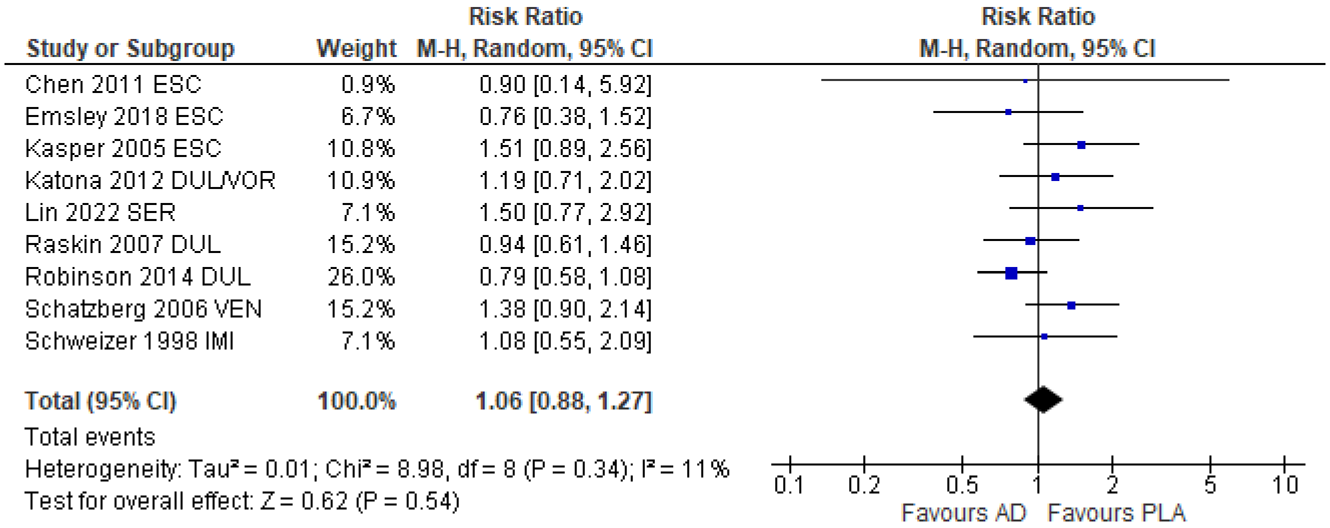

Table S1. PRISMA for Pairwise Meta-Analyses Checklist.

Section and Topic	Item #	Checklist item	Location where item is reported
**TITLE**			
Title	1	Identify the report as a systematic review.	P1
**ABSTRACT**			
Abstract	2	See the PRISMA 2020 for Abstracts checklist.	P3
**INTRODUCTION**			
Rationale	3	Describe the rationale for the review in the context of existing knowledge.	P4
Objectives	4	Provide an explicit statement of the objective(s) or question(s) the review addresses.	P4
**METHODS**			
Eligibility criteria	5	Specify the inclusion and exclusion criteria for the review and how studies were grouped for the syntheses.	P5
Information sources	6	Specify all databases, registers, websites, organisations, reference lists and other sources searched or consulted to identify studies. Specify the date when each source was last searched or consulted.	P5
Search strategy	7	Present the full search strategies for all databases, registers and websites, including any filters and limits used.	P5
Selection process	8	Specify the methods used to decide whether a study met the inclusion criteria of the review, including how many reviewers screened each record and each report retrieved, whether they worked independently, and if applicable, details of automation tools used in the process.	P5
Data collection process	9	Specify the methods used to collect data from reports, including how many reviewers collected data from each report, whether they worked independently, any processes for obtaining or confirming data from study investigators, and if applicable, details of automation tools used in the process.	P5
Data items	10a	List and define all outcomes for which data were sought. Specify whether all results that were compatible with each outcome domain in each study were sought (e.g. for all measures, time points, analyses), and if not, the methods used to decide which results to collect.	P5
	10b	List and define all other variables for which data were sought (e.g. participant and intervention characteristics, funding sources). Describe any assumptions made about any missing or unclear information.	P5
Study risk of bias assessment	11	Specify the methods used to assess risk of bias in the included studies, including details of the tool(s) used, how many reviewers assessed each study and whether they worked independently, and if applicable, details of automation tools used in the process.	P5
Effect measures	12	Specify for each outcome the effect measure(s) (e.g. risk ratio, mean difference) used in the synthesis or presentation of results.	P5
Synthesis methods	13a	Describe the processes used to decide which studies were eligible for each synthesis (e.g. tabulating the study intervention characteristics and comparing against the planned groups for each synthesis (item #5)).	P5
	13b	Describe any methods required to prepare the data for presentation or synthesis, such as handling of missing summary statistics, or data conversions.	P5
	13c	Describe any methods used to tabulate or visually display results of individual studies and syntheses.	P5
	13d	Describe any methods used to synthesize results and provide a rationale for the choice(s). If meta-analysis was performed, describe the model(s), method(s) to identify the presence and extent of statistical heterogeneity, and software package(s) used.	P5
	13e	Describe any methods used to explore possible causes of heterogeneity among study results (e.g. subgroup analysis, meta-regression).	P5
	13f	Describe any sensitivity analyses conducted to assess robustness of the synthesized results.	P5
Reporting bias assessment	14	Describe any methods used to assess risk of bias due to missing results in a synthesis (arising from reporting biases).	P5

Section and Topic	Item #	Checklist item	Location where item is reported
Certainty assessment	15	Describe any methods used to assess certainty (or confidence) in the body of evidence for an outcome.	P5
**RESULTS**			
Study selection	16a	Describe the results of the search and selection process, from the number of records identified in the search to the number of studies included in the review, ideally using a flow diagram.	P6
	16b	Cite studies that might appear to meet the inclusion criteria, but which were excluded, and explain why they were excluded.	P6
Study characteristics	17	Cite each included study and present its characteristics.	P6
Risk of bias in studies	18	Present assessments of risk of bias for each included study.	P6
Results of individual studies	19	For all outcomes, present, for each study: (a) summary statistics for each group (where appropriate) and (b) an effect estimate and its precision (e.g. confidence/credible interval), ideally using structured tables or plots.	P6
Results of syntheses	20a	For each synthesis, briefly summarise the characteristics and risk of bias among contributing studies.	P6
	20b	Present results of all statistical syntheses conducted. If meta-analysis was done, present for each the summary estimate and its precision (e.g. confidence/credible interval) and measures of statistical heterogeneity. If comparing groups, describe the direction of the effect.	P6
	20c	Present results of all investigations of possible causes of heterogeneity among study results.	P6
	20d	Present results of all sensitivity analyses conducted to assess the robustness of the synthesized results.	P7
Reporting biases	21	Present assessments of risk of bias due to missing results (arising from reporting biases) for each synthesis assessed.	P6
Certainty of evidence	22	Present assessments of certainty (or confidence) in the body of evidence for each outcome assessed.	P6
**DISCUSSION**			
Discussion	23a	Provide a general interpretation of the results in the context of other evidence.	P8
	23b	Discuss any limitations of the evidence included in the review.	P8
	23c	Discuss any limitations of the review processes used.	P8
	23d	Discuss implications of the results for practice, policy, and future research.	P8
**OTHER INFORMATION**			
Registration and protocol	24a	Provide registration information for the review, including register name and registration number, or state that the review was not registered.	P5
	24b	Indicate where the review protocol can be accessed, or state that a protocol was not prepared.	P5
	24c	Describe and explain any amendments to information provided at registration or in the protocol.	P5
Support	25	Describe sources of financial or non-financial support for the review, and the role of the funders or sponsors in the review.	P10
Competing interests	26	Declare any competing interests of review authors.	P9
Availability of data, code and other materials	27	Report which of the following are publicly available and where they can be found: template data collection forms; data extracted from included studies; data used for all analyses; analytic code; any other materials used in the review.	P11

From: Page MJ, McKenzie JE, Bossuyt PM, Boutron I, Hoffmann TC, Mulrow CD, et al. The PRISMA 2020 statement: an updated guideline for reporting systematic reviews. BMJ 2021;372:n71. doi: 10.1136/bmj.n71

For more information, visit: http://www.prisma-statement.org/

Table S2. Efficacy results of the original study and the data synthesis of our meta-analysis.

Study	Results of the study †	The definition of response	Depression scale ‡	Results of the study †	The definition of response	Results of the study †	The definition of remission	Results of the study*
Chen 2011	ESC > PLA	Cure and considerable improvement	GDS	ESC > PLA	Cure and improvement	No statistical results were presented.	NR	
Emsley 2018	ESC > PLA	≥ 50% improvement in HAMD17	HAMD17	ESC > PLA	≥ 50% improvement in HAMD17	ESC > PLA	NR	
Kasper 2005	ESC = PLA	≥ 50% improvement in MADRS	MADRS	ESC = PLA	≥ 50% improvement in MADRS	ESC = PLA	MADRS ≤ 12	ESC = PLA
Katona 2012	DUL > PLA<br>VOR > PLA	≥ 50% improvement in MADRS	HAMD24	DUL > PLA<br>VOR > PLA	≥ 50% improvement in MADRS	DUL > PLA<br>VOR > PLA	HAMD17 ≤ 7	DUL > PLA<br>VOR > PLA
Lin 2022	SER = PLA	NR	HAMD17	SER = PLA	NR		NR	
Raskin 2007	DUL > PLA	≥ 50% improvement in HAMD17	HAMD17	DUL > PLA	≥ 50% improvement in HAMD17	DUL > PLA	HAMD17 ≤ 7	DUL > PLA
Robinson 2014	DUL = PLA	≥ 50% improvement in HAMD17	HAMD17	DUL = PLA	≥ 50% improvement in HAMD17	No statistical results were presented.	HAMD17 ≤ 7	No statistical results were presented.
Schatzberg 2006	VEN = PLA	≥ 50% improvement in HAMD17	HAMD21	VEN = PLA	≥ 50% improvement in HAMD17	VEN = PLA	HAMD17 ≤ 7	VEN = PLA
Schweizer 1998	IMI > PLA	≥ 50% improvement in HAMD17	HAMD17	IMI > PLA	≥ 50% improvement in HAMD17	IMI > PLA	NR	

† A = B: A was similar to B, A > B: A was superior to B.

‡ When standard errors instead of standard deviations (SD) were presented, the former was converted to standard deviations (SDs). If both were missing, we estimated SDs from confidence intervals, t-values, or p-values as described in Section 7.7.3 of the Cochrane Handbook for Systematic Reviews. If none of these options was viable, we contacted the original authors. When no information could be obtained, we derived SDs from those of the other studies using a validated imputation technique.

Huhn M, Nikolakopoulou A, Schneider-Thoma J, Krause M, Samara M, Peter N, Arndt T, Bäckers L, Rothe P, Cipriani A, Davis J, Salanti G, Leucht S. Comparative efficacy and tolerability of 32 oral antipsychotics for the acute treatment of adults with multi-episode schizophrenia: a systematic review and network meta-analysis. Lancet. 2019 Sep 14;394(10202):939-951.

DUL: duloxetine, ESC: escitalopram, GDS: Geriatric Depression Scale, HAMD: Hamilton Depression Rating Scale, IMI: imipramine, MADRS: Montgomery Åsberg Depression Rating Scale, NR: not report, PLA: placebo, SER: sertraline, VEN: venlafaxine, VOR: vortioxetine

Table S3. Characteristics of randomized controlled trials in our systematic review and meta-analysis.

	Duration (week)	Diagnosis	Minimum severity	Medication before the trial	Patient status	Country	Concomitant medication	Drug	Dose (mg/d)	Dosing schedule	n	Age (mean±SD)	%male	Severity at BL (mean±SD)
Chen 2011	8	DSM-IV-TR	GDS>20	None	NR	China	NR	ESC	10	Fixed	29	68.9 ± 6.1	38.2	23.4 ± 4.7 (GDS)
								PLA			26			24.0 ± 4.70(GDS)
Emsley 2018	8	DSM-IV-TR	HAMD17≥22, CGI (item 1) ≥4, HAD (dep)≥11	Washout before starting the trial	OP	International	Allow (BEN, ZOL, ZOP)	ESC	10	Fixed	99	70.3±4.9	25.3	26.7±3.2 (HAMD17)
								PLA			107	70.8±5.1	25.2	26.6±3.5 (HAMD17)
Kasper 2005	8	DSM-IV	MADRS≥22-≤40, MMSE≥22	Washout during screening and PLA lead-in phase	IP and OP	International	Allow (OXA, TEM, ZOP, ZOL)	ESC	10	Fixed	173	75±7	74.7	28.2±3.8 (MADRS)
								PLA			180	75±7	23.8	28.6 ±4.2 (MADRS)
Katona 2012	8	DSM-IV-TR	MADRS≥26, MMSE≥24	Yes	NR	International	Allow	DUL	60	Fixed	151	70.9±5.5	33.8	30.4±3.1 (MADRS)
								VOR	5	Fixed	156	70.5±4.8	31.4	30.7±3.6 (MADRS)
								PLA			145	70.3±4.4	37.9	30.3±3.2 (MADRS)
Lin 2022	8	DSM-IV	HAMD17≥18, MMSE≥20	None	NR	Taiwan	Allow (LOR)	SER	25-150[†]	Flexible	39	66.9±7.2	12.8	27.0±5.2 (HAMD17)
								PLA			39	70.5±7.3	25.6	25.4±6.0 (HAMD17)
Raskin 2007	8	DSM-IV	HAMD17≥18, MMSE≥20	Washout during screening and PLA lead-in phase	OP	USA	Allow	DUL	60	Fixed	207	72.6±5.7	39.6	22.7±7.0 (HAMD17)
								PLA			104	73.3±5.7	42.3	23.2±7.0 (HAMD17)
Robinson 2014	12	DSM-IV-TR	MADRS≥20, MMSE≥20	Washout during PLA lead-in phase	OP	International	NR	DUL	60	Fixed	249	73.0±6.3	33.8	19.4±5.6 (HAMD17)
								PLA			121	73.1±5.6	41.1	19.3±5.8 (HAMD17)
Schatzberg 2006	8	DSM-IV	HAMD21≥20, MMSE≥19	None	OP	USA	Allow (CHL, ZOL)	VEN	150-225	Flexible	104	71	46.2	24 (HAMD17)
								PLA			96	71	52.1	23 (HAMD17)
Schweizer 1998	8	DSM-III-TR	HAMD17≥18, MMSE≥20	None	OP	USA	Not allow	IMI	25-150	Flexible	60	72±6.7[‡]	70.1[‡]	23.9±4.0 (HAMD17)
								PLA			60			24.1±4.2 (HAMD17)

† Final mean dose: 66.7 ± 30.5 mg/d

‡ Data from all participants (buspirone, imipramine, and placebo arm)

BEN: benzodiazepine, BL: baseline, CGI: Clinical Global Impressions, CHL: chloral hydrate, d: day, DSM(-TR): Diagnostic and Statistical Manual of Mental Disorders (-Text Revision), DUL: duloxetine, ESC: escitalopram, GDS: Geriatric Depression Scale, HAD: Hospital Anxiety and Depression scale, HAMD: Hamilton Depression Rating Scale, IMI: imipramine, IP: inpatient, LOR: lorazepam, MADRS: Montgomery Åsberg Depression Rating Scale, MMSE: Mini-Mental State Examination, n: number of individuals, NR: not report, OP: outpatient, OXA: oxazepam, PLA: placebo, SD: standard deviation, SER: sertraline, TEM: temazepam, USA: United States of America, VEN: venlafaxine, VOR: vortioxetine, ZOL: zolpidem, ZOP: zopiclone

Table S4. Sensitivity analysis for the primary outcome.

		K (n)	RR (95% CI)	I^2	Test for subgroup differences
Antidepressant class	Newer antidepressants studies	7 (1842)	1.35 (0.98, 1.84)	84 %	P = 0.36, I^2 = 0%
	Older antidepressant study†	1 (118)	1.70 (1.15, 2.53)	na	
Antidepressant class‡	SSRI studies	3 (605)	1.47 (0.76, 2.87)	85%	P= 0.65, I^2 = 0%
	SNRI studies	4 (1083)	1.36 (0.86, 2.13)	88%	
	Vortioxetine study	1 (299)	1.67 (1.29, 2.15)	na	
Overall risk of bias	Some concern and low risk	7 (1909)	1.34 (1.03, 1.74)	82%	P= 0.03, I^2 = 79.9%
	High risk	1 (51)	31.25 (1.98, 493.21)	na	

†The older antidepressant subgroup included only imipramine.

‡The older antidepressant subgroup only included imipramine. The SSRI subgroup only included escitalopram. For the Katona 2012 study in this subgroup analysis, we used the data separately for the duloxetine and vortioxetine arms.

na: not applicable, SNRI: Serotonin Noradrenaline Reuptake Inhibitor, SSRI: Selective Serotonin Reuptake Inhibitor

Table S5. Meta-regression analysis for the primary outcome.

	Coefficient	Standard error	95% CI	Z	p	I^2
Mean age	-0.126	0.079	-0.281, 0.029	-1.598	0.110	73.46%
%male	-0.003	0.011	-0.025, 0.020	-0.258	0.796	83.18%
Antidepressant dose †	-0.495	0.485	-1.445, 0.456	-1.020	0.308	82.40%
Dosing schedule	-0.128	0.342	-0.799, 0.543	-0.374	0.708	83.84%
Study duration	-0.122	0.097	-0.313, 0.069	-1.256	0.209	79.30%
Total participants	-0.001	0.001	-0.004, 0.002	-0.637	0.524	83.90%

†Defined daily dose (https://www.who.int/tools/atc-ddd-toolkit/about-ddd)

For the imipramine dose in the Schatzberg 2006 study, we used the median dose of imipramine used during the study.

Table S6. Sensitivity analysis excluding the imipramine study.

	The primary meta-analysis		This sensitivity analysis	
	RR (95% CI)	I^2	RR (95% CI)	I^2
All-cause discontinuation	1.06 (0.88, 1.27)	11%	1.07 (0.87, 1.32)	22%
Discontinuation due to adverse events	1.94 (1.30, 2.88)	25%	2.04 (1.37, 3.02)	24%
At least one adverse event	1.11 (1.02, 1.21)	51%	1.09 (0.99, 1.20)	51%

Imipramine study did not report data of remission rate.
